# Early signs of multi-walled carbon nanotbues degradation in macrophages, via an intracellular pH-dependent biological mechanism; importance of length and functionalization

**DOI:** 10.1186/s12989-016-0175-z

**Published:** 2016-11-24

**Authors:** Marion Landry, Mathieu Pinault, Stéphane Tchankouo, Émeline Charon, Audrey Ridoux, Jorge Boczkowski, Martine Mayne-L’Hermite, Sophie Lanone

**Affiliations:** 1 INSERM, U955, Equipe 4, Créteil, F-94000 France; 2 NIMBE, CEA, CNRS, Université Paris-Saclay, CEA Saclay, 91191 Gif sur Yvette Cedex, France; 3 Université Paris Est-Créteil, Faculté de Médecine de Créteil, 8 rue du Général Sarrail, Créteil, F-94000 France; 4 DHU A-TVB, Service d’explorations fonctionnelles respiratoires, Assistance Publique Hôpitaux de Paris, Hôpitaux Universitaires Henri Mondor, Créteil, F-94000 France

**Keywords:** Carbon nanotubes, Biodegradation, pH, Functionalization, Length

## Abstract

**Background:**

Carbon nanotubes (CNT) can interact with the biological environment, which could participate in their associated toxicity. We recently demonstrated that pH is an important player of CNT fate inside macrophages. We wanted to further characterize such process, and therefore designed a study dedicated to decipher CNT biodegradation by macrophages, as a function of two major physico-chemical properties in regard with nanotoxicology; length and degree of functionalization. To achieve our aim, we synthesized, following a single initial production process, four MWCNT differing in length and/or surface chemistry: S-CNT (short), SF-CNT (short functionalized), L-CNT (long) and LF-CNT (long functionalized).

**Results:**

Raman spectroscopy analysis performed on CNT recovered after exposure of RAW 264.7 macrophages for 6, 24, or 48 h demonstrate that CNT show early signs of biodegradation over time inside macrophages. The modulation of CNT length and functionalization, resulting in the modification of iron accessibility, both represent critical determinants of the biodegradation process; short pristine CNT were more prone to biodegradation than long CNT (pristine or functionalized), while short functionalized CNT were protected. Incubation of cells with Concanamycin completely prevents CNT from being modified, demonstrating that this biodegradation process is dependent on an intracellular pH-dependent mechanism. Interestingly, and despite evidence of degradation via Raman spectroscopy, the CNT length and diameter were not altered during the course of the study.

**Conclusions:**

In conclusion, our results identify a new mechanism of CNT biodegradation inside macrophages. This could give new insights for the understanding of CNT-associated toxicity, and represent important tools to develop safe(r)-by-design nanomaterials.

**Electronic supplementary material:**

The online version of this article (doi:10.1186/s12989-016-0175-z) contains supplementary material, which is available to authorized users.

## Background

Carbon nanotubes (CNT) are a family of nanomaterials featuring unique properties and presenting a large range of length, diameter, and number of walls (single-walled -SWCNT-, or multi-walled -MWCNT-). The current applications for SWCNT range from chemical sensors, to conductive heating films, conductive nanoink, nanodevice or display. These applications need high quality but small amount of CNT, whereas those utilizing MWCNT as conducting paints, composite materials (heat exchanger, reinforced materials, …) or developed for energy storage require large amounts of CNT [1–4]. Due to this expansion of MWCNT usage and the resulting likely increase of human exposure, the potential adverse effects of CNT, and particularly those of MWCNT on human health are of great concern. A large body of literature indicates that CNT can be toxic, depending on numerous physicochemical characteristics including length, diameter, structural defects, surface area, tendency to agglomerate, dispersibility in solution, presence and nature of catalyst residues, as well as surface chemistry (see [5] for review).

It is now well accepted that nanomaterials, including MWCNT, can interact with the biological environment, which could participate in their associated toxicity [6]. This is demonstrated by the rapid formation of a bio-corona (proteins, lipids, other biomolecules) around the nanomaterials, which potentially confers them new or different (surface) identity. More recently, data from the literature also evidenced that SWCNT can undergo enzymatic biodegradation. So far, the majority of the studies dedicated to evaluate the degradation of CNT have been conducted with SWCNT in a-cellular systems supplemented with recombinant enzymes. Indeed, recent reports have demonstrated that the plant horseradish peroxidase (HRP), and the animal peroxidases myeloperoxidase (MPO) and eosinophil peroxidase (EPO) are able to catalyze the degradation of CNT [7–12]. However, because of their experimental set-up far from real-life exposure (a-cellular systems, use of recombinant enzymes, high doses of H_2_O_2_ to catalyze the reaction, …), these studies, although informative, remain of limited impact in the context of human health. More recently, a few studies have been conducted in cell cultures, demonstrating that SWCNT can be biodegraded inside most inflammatory cell types (neutrophils, eosinophils or monocytic cells), via a MPO- or EPO-dependent mechanism [8, 13, 14]. Surprisingly, despite the unique role of macrophages in the in vivo elaboration of CNT-induced inflammation [15], almost no study so far have been conducted to evaluate CNT biodegradation in this cell type [14, 16, 17]. Such evaluation could be particularly relevant since it is long known that CNT-loaded macrophages are present at the site of exposure and/or distributed throughout the body, up to 24 months after the initial exposure to CNT [18]. Moreover, these studies were most exclusively conducted with SWCNT although they do not represent the majority of the CNT produced, and therefore might not represent the major risk for human exposure. Finally, another aspect that is currently absent in the literature is the importance of CNT physico-chemical characteristics in their biodegradation, although, as major determinants of CNT toxicity, one can imagine that these characteristics might largely influence CNT biodegradation as well [5].

We therefore developed a study dedicated to characterize the biodegradation of MWCNT by macrophages, as a function of three major physico-chemical properties in regard with nanotoxicology; their length, degree of functionalization and their iron-based catalyst residual content [5]. To achieve our aim, we synthesized, following a dedicated initial process, four MWCNT specifically devoted to our study and differing in length, and that were functionalized to concomitantly modify their surface chemistry and/or iron content (Additional file 1: Figure S1): S-CNT (short), SF-CNT (short and functionalized), L-CNT (long) and LF-CNT (long and functionalized), functionalized CNT (SF- and LF-CNT) having a different iron content from their non-functionalized counterpart (S- and L-CNT) (Table 1). The biodegradation of these CNT was addressed in RAW 264.7 murine macrophages in vitro, by the mean of Raman spectroscopy performed on CNT recovered in cells, after 3 different exposure time points; 6, 24 or 48 h. Moreover, as we recently demonstrated that pH could be an important player in the fate of CNT inside cells [19], we also investigated the role of pH in CNT biodegradation process. Our results demonstrate that MWCNT can be biodegraded inside macrophages, in a time-dependent manner, via an intracellular pH-dependent biological mechanism. The modulation of CNT length and functionalization, concomitantly resulting in the modification of iron accessibility, both represent critical determinants of the biodegradation process.

**Table 1:** Characteristics of the CNT

	S-CNT	SF-CNT	L-CNT	LF-CNT
Diameter, mean (nm)	26	25	24	25
Length, mean ± SD (μm)	1.7 ± 0.9	1.6 ± 1.6	6.4 ± 4.3	9.2 ± 5.8
- CNT < 2 μm (%)	69	74.5	2.5	0
- CNT < 5 μm (%)	100	99.5	51	23
- CNT < 10 μm (%)	100	100	84	67
- CNT < 20 μm (%)	100	100	98	93
Iron content (TGA), mean (weight %)	4.4	1.3	4.5	1.0
Composition (XPS)				
Carbon (atomic %)	98.6	91.7	97.2	91.6
Oxygen (atomic %)	1.4	8.1	1.8	8.3
Sulfur (atomic %)	0	0.2	0	0.1
Endotoxin levels	ND	ND	ND	ND
Intrinsic ROS production, mean ± SD	0.92 ± 0.01	0.05 ± 0.01	0.77 ± 0.05	0.65 ± 0.05

*SD* standard deviation, *ND* not detectable

## Results

### CNT characterization

**Fig. 1:**
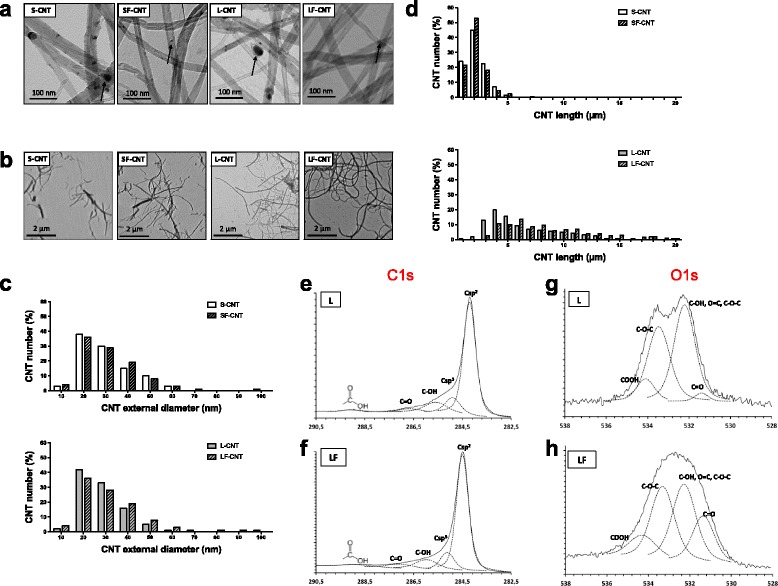
Microscopy images and XPS analysis of CNT powders. Panel **a** Transmission electron microscope (TEM) images of S-, SF-, L- and LF-CNT. Scale bar: 100 nm. Panel **b** Optical microscope images of S-, SF-, L- and LF-CNT. Scale bar: 2 μm. Panel **c** CNT external diameter distribution. Panel **d** CNT length distribution. Typical spectra of the C1s (Panel **e** and **f**) and O1s core level (Panel **g** and **h**) obtained for L- (Panel **e** and **g**) or LF-CNT (Panel **f** and **h**). S-CNT: short-carbon nanotubes; SF-CNT: short functionalized carbon nanotubes; L-CNT: long carbon nanotubes; LF-CNT: long functionalized carbon nanotubes

The main physicochemical characteristics of CNT, deduced from the comprehensive analysis presented below, are summarized in Table 1. Transmission Electron Microscopy (TEM) and Optical Microscopy (OM) images of the initial CNT carpets are presented in Fig. 1a and b. TEM observations showed that all CNT batches contained iron-based particles either attached at their external basis and encapsulated in carbon sheets, or mainly entrapped inside their hollow core (Fig. 1a). The four CNT batches showed similar mean external diameter (Table 1) and distribution (Fig. 1c); neither the shortening nor the acidification treatment modified the external diameter of the samples. Mean length was measured at 1.7 and 1.6 μm for S- and SF-CNT respectively, and at 6.4 and 9.2 μm for L- and LF-CNT respectively (Table 1). Length distribution for S- and SF-CNT on one hand, and L- and LF-CNT on the other hand, was similar (Fig. 1d). Thermogravimetric analysis (TGA) showed that the average iron content was similar for S- and L-CNT (4.4 and 4.5 wt% respectively), and for SF- and LF-CNT (1.3 and 1.0 wt% respectively, Table 1). X-ray-induced photoelectron spectroscopy (XPS) analysis revealed a higher content of oxygen atoms (8.1 at% and 8.3 at% respectively) for SF- and LF-CNT as compared to S- and L-CNT (1.4 at% and 1.8 at% respectively), consistent with the functionalization by acid treatment and the presence of various functions (mainly: C-OH, C = O and O-C-O in different possible groups e.g. carboxylic, anhydride, ester) at the surface of CNT (Table 1 and Fig. 1e-h). Moreover, SF- and LF-CNT showed traces of sulfur, most likely due to the use of sulfuric acid during the acid treatment. No detectable endotoxin content could be detected regardless of the CNT. Finally, measurement of the intrinsic ROS production showed higher content for S-CNT as compared to L- and LF-CNT, and no/very low ROS production could be detected for SF-CNT.

**Fig. 2:**
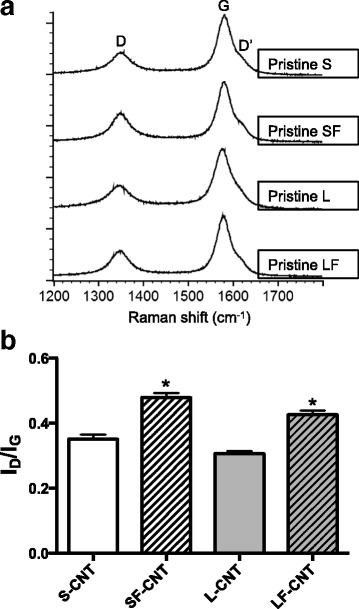
Raman spectroscopy analysis of CNT powders. Panel **a** Typical Raman spectra obtained on S-, SF-, L-, and LF-CNT. Panel **b** I_D_/I_G_ ratio calculated from Raman spectra of S-, SF-, L-, and LF-CNT powders. Data are given as mean ± SEM of minimum 5 values. *: *p* < 0.05 vs non-functionalized counterpart. Abbreviations as in Fig. 1

Typical Raman spectra, that reflect the degree of defects in CNT, are given in Fig. 2a. The calculated I_D_/I_G_ ratio (Fig. 2b) appears higher for the functionalized (0.55 and 0.42 for SF- and LF-CNT respectively) as compared to the non-functionalized CNT (0.35 and 0.31 for S- and L-CNT respectively, *p* < 0.05), which indicates that the grafting of chemical species involves also the formation of defects in the graphene carbonaceous structure as often reported in the literature [20].

### CNT are modified inside macrophages

**Fig. 3:**
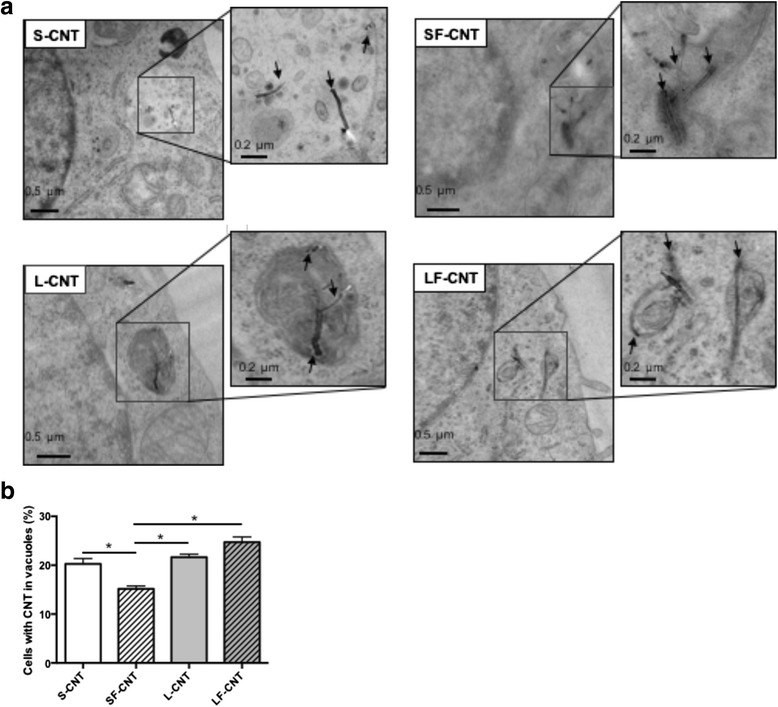
TEM images of RAW 264.7 macrophages exposed to CNT for 6 h. Panel **a** Typical TEM images of RAW 264.7 cells exposed to CNT for 6 h. Panel **b** quantification of the percentage of cells presenting CNT inside phagocytic vacuoles. Abbreviations as in Fig. 1

**Fig. 4:**
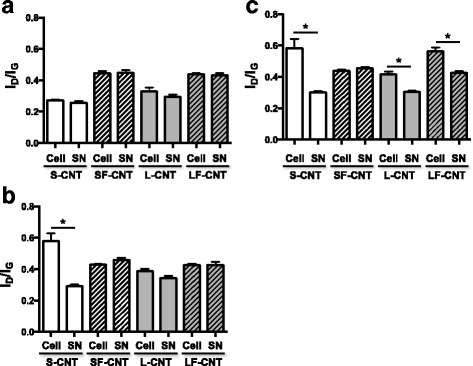
Raman spectroscopy analysis of CNT recovered from cell cultures. I_D_/I_G_ ratio calculated from Raman spectra of S-, SF-, L-, or LF-CNT recovered from cellular (Cell) or supernatant (SN) fractions of RAW 264.7-exposed macrophages for 6 (Panel **a**), 24 (Panel **b**) or 48 h (Panel **c**). Data are given as mean ± SEM of minimum 5 values. Abbreviations as in Fig. 1. *: *p* < 0.05

**Fig. 5:**
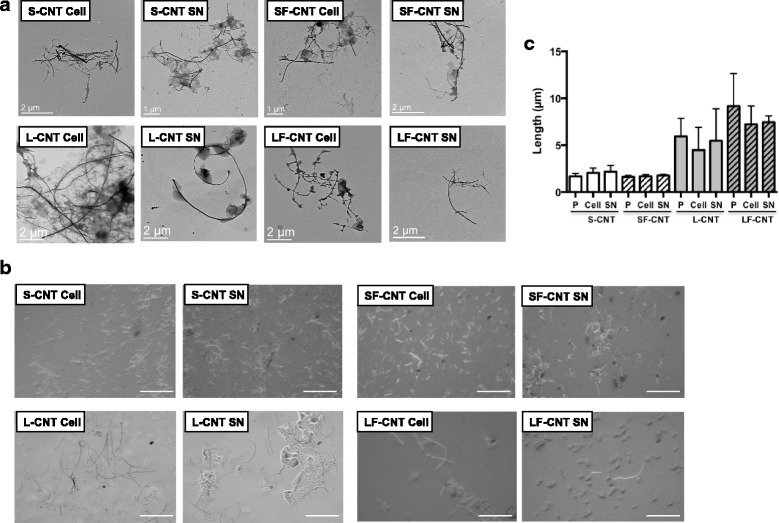
Microscopy images of CNT after RAW 264.7 macrophages exposure for 48 h. Panel **a** TEM images of S-, SF-, L- and LF-CNT recovered in RAW 264.7 cells exposed for 48 h to the different CNT. Panel **b** Optical microscopy images of S-, SF-, L- and LF-CNT recovered in RAW 264.7 cells exposed for 48 h to the different CNT. Panel **c** Length of S-, SF-, L-, and LF-CNT recovered from the cellular (Cell) or supernatant (SN) fractions of 48 h-exposed RAW 264.7. Abbreviations as in Fig. 1. Data are given as mean ± SEM

Figure 3a shows representative transmission electronic microscopy (TEM) images of macrophages exposed to CNT for 6 h. All CNT could be internalized by macrophages, mainly inside vacuoles although to a lesser extent for SF-CNT (Fig. 3b). Whatever the CNT, images showing CNT penetrating through the vacuole wall could also been observed. To address the issue of a potential modification of CNT while incorporated inside the cells, the cellular (Cell) and supernatant (SN) fractions of macrophage cultures were recovered and separately analyzed after exposure of RAW 267.4 macrophages during 6, 24 or 48 h. As shown in Fig. 4a, I_D_/I_G_ ratio obtained 6 h after the initial exposure were similar to those obtained for CNT powders (Fig. 2), and no difference was observed between the different fractions considered (Cell or SN). However, starting from 24 h for S- and 48 h for L- and LF-CNT, we could observe a significant increase of the I_D_/I_G_ ratio in the cellular fraction only as compared to the supernatant one (0.58 ± 0.05 and 0.29 ± 0.01 for SCNT-Cell and SCNT-SN respectively after 24 h -Fig. 4b, and, after 48 h, 0.58 ± 0.06 and 0.3 ± 0.01 for SCNT-Cell and SCNT-SN respectively, 0.42 ± 0.02 and 0.3 ± 0.01 for LCNT-Cell and LCNT-SN respectively and 0.56 ± 0.02 and 0.43 ± 0.01 for LFCNT-Cell and LFCNT-SN respectively - Fig. 4c, and Additional file 2: Figure S2, Cell versus SN, *p* < 0.05). Whatever the time point and CNT studied, the I_D_/I_G_ ratio obtained in SN fractions were similar to those of the original powders of CNT (Fig. 2). In addition, no significant modification of CNT length could be detected in Cell or SN fractions of RAW macrophages exposed up to 48 h, regardless of the CNT used (Fig. 5).

### CNT modifications are driven by an intracellular pH-dependent mechanism

**Fig. 6:**
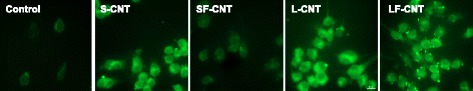
Lysosomal activity assessment. Lysosensor assay in RAW 264.7 macrophages exposed to CNT for 6 h. Scale bar: 10 μm. Abbreviations as in Fig. 1

As previous studies from our laboratory demonstrate the role of pH in the detachment of iron catalyst nanoparticles from SWCNT and subsequent toxicity [19], we next examined intracellular pH modifications in RAW 264.7 macrophages exposed to the various CNT. As shown in Fig. 6, all but SF-CNT induced the acidification of lysosomal compartment after 6 h exposure. Such acidification remains detectable, although faintly, only in S-CNT exposed macrophages after 24 h, and was no more observed after 48 h treatment (Additional file 3: Figure S3).

**Fig. 7:**
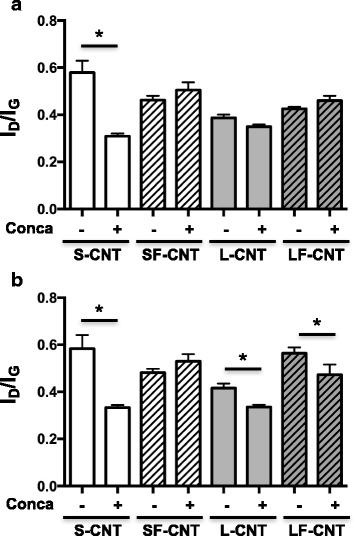
Raman spectroscopy analysis of CNT recovered from cells cultured in presence or absence of Concanamycin. I_D_/I_G_ ratio calculated from Raman spectra of S-, SF-, L-, or LF-CNT recovered from RAW 264.7 macrophages concomitantly exposed to CNT and Concanamycin A for 24 h (Panel **a**) or 48 h (Panel **b**). Abbreviations as in Fig. 1. Conca: Concanamycin A. Data are given as mean ± SEM of minimum 5 values

In order to assess the role of intracellular pH in the observed biodegradation of CNT, Raman spectroscopy was performed in the cellular fraction (Cell) of macrophages exposed to CNT in presence of the H^+^-ATPase inhibitor Concanamycin A. As shown in Fig. 7, incubation of cells with Concanamycin completely prevents CNT from being modified, suggesting that CNT modifications were the result of an intracellular pH-dependent mechanism. This is also confirmed by observations showing no modification of Raman spectra in the supernatant fraction (SN) of macrophages exposed to CNT in presence of Concanamycin, irrespective of the time point and CNT considered (data not shown).

**Fig. 8:**
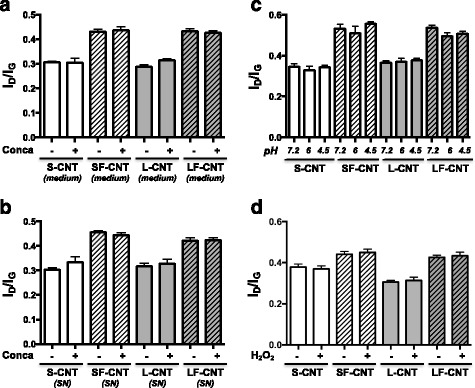
Raman spectroscopy analysis of CNT incubated in media presenting various acidity. Panel **a** I_D_/I_G_ ratio calculated from Raman spectra of S-, SF-, L-, or LF-CNT incubated for 48 h in culture medium in presence or absence of Concanamycin A. Panel **b** I_D_/I_G_ ratio calculated from Raman spectra of S-, SF-, L-, or LF-CNT incubated for 48 h in the supernatant of cells cultured in presence or absence of Concanamycin A. Panel **c** I_D_/I_G_ ratio calculated from Raman spectra of S-, SF-, L-, or LF-CNT incubated for 48 h in artificial medium which pH was set-up at 7.2, 6, or 4.5. Panel **d** I_D_/I_G_ ratio calculated from Raman spectra of S-, SF-, L-, or LF-CNT incubated for 48 h in culture medium in presence or absence of H_2_O_2_. Abbreviations as in Fig. 1. Data are given as mean ± SEM of minimum 5 values

The protection against CNT biodegradation brought by Concanamycin treatment could result, beside from pH-dependent mechanism driven by an intracellular event, from 3 different events 1/ a direct effect of Concanamycin on CNT structure, 2/ an effect of Concanamycin on cell secretome which could, in turn, modify CNT structure, or, finally, 3/ a direct effect of pH on CNT. The experiments performed to test these 3 last hypotheses demonstrate that none of them were correct. Indeed, CNT Raman spectra are not modified when CNT were incubated for 48 h 1/ in culture medium supplemented with Concanamycin (Fig. 8a), 2/ in supernatant from cells treated with Concanamycin (Fig. 8b), or 3/ in an artificial solution representative of lysosomal compartments (Fig. 8c). Finally, incubation of CNT with H_2_O_2_ for 48 h didn’t induce any modification in Raman spectra (Fig. 8d). Overall, these results are the first evidence of the degradation of CNT inside the cells *via* an intracellular pH-dependent mechanism.

## Discussion

Overall, our data demonstrate that CNT can be biodegraded over time inside macrophages, with a combined influence of both CNT length and acidic functionalization; short pristine CNT were more prone to biodegradation than long CNT (pristine or functionalized), while short functionalized CNT were protected. Moreover, we showed that this biodegradation process is dependent on an intracellular pH-dependent mechanism, and not associated to a modification of CNT length or diameter that could be detected during the time course of the study.

All but SF-CNT show early signs of degradation in macrophages, starting at 24 h for S-CNT, and 48 h for L- and LF-CNT. Pretreatment of cells with the V-ATPase inhibitor Concanamycin protects all CNT from their biodegradation by macrophages, which strongly suggests that this biodegradation process is pH-dependent. The exclusive intracellular localization of this pH-dependent mechanism is also strongly suggested by the absence of Concanamycin effect on CNT structure (directly or by the mean of cellular secretome) or any direct effect of pH on CNT. To the best of our knowledge, this is the first evidence of such a specifically intracellular-driven mechanism as the few studies studying CNT degradation in cellular conditions have been performed on samples containing both cells and supernatant together, which could lead to a confusion on the origin of the biodegradation process [8, 13, 14]. Indeed, thanks to our experimental protocol, we were able to isolate CNT present inside the cells from those present in the supernatant of the exposed cells. The pH-dependence of CNT biodegradation process is also confirmed by the fact that the only CNT batch that was not biodegraded (SF-CNT) was also the only one that did not induce an intracellular acidification after 6 h. This could be related to the lesser internalization of SF-CNT inside phagocytic vacuoles as compared to the other batches of CNT. Indeed, it has been described that, in macrophages, phagocytosis is rapidly accompanied by the recruitment of V-ATPases to the phagosome membrane, leading to a rapid decrease of pH [21]. We recently showed that intracellular acidification of macrophages leads to the detachment of iron-based catalyst nanoparticles initially attached to CNT [19]. This accessible iron could react, *via* the Fenton reaction, and, in turn, lead to the generation of ROS, which are proposed to be important mediators of CNT degradation [5]. Interestingly, the overall biodegradation of CNT in our experimental set-up strictly followed the intrinsic ROS production by CNT *per se*, with high levels for S-CNT, lower (and similar) levels for L- and LF-CNT, and absence of ROS production by SF-CNT. Recent studies have proposed peroxidases, and most frequently myeloperoxidase (MPO), as potential candidate for the biodegradation of SWCNT [7, 8, 13, 22, 23], together with reactive intermediates that are considered to be key factors for SWCNT degradation by cells. However, macrophages are relatively poor in MPO or NADPH oxidases (NOX), and both MPO and NOX are inactive at acidic pH, which rules out their potential role in the observed biodegradation process at least in the time-course of our study [24–26]. Finally, and beside a direct reactivity of CNT surface because of the acidic environment, this pH-dependent intracellular mechanism could rely on protein neosynthesis (or a cascade of protein synthesis) or other biological actions triggered by acidic pH (activation of acidic hydrolases, …). Indeed, given the delay between cellular acidification (present at 6 h but no more or only faintly for S-CNT after 24 h) and the first observable signs of CNT biodegradation, the intracellular acidification that occurs in response to CNT might be the initial event driving their subsequent surface modifications. Such mechanisms however deserve further studies to be fully elucidated.

S-CNT were the more susceptible to biodegradation as compared to L- and LF-CNT, both in terms of kinetics (modifications visible as soon as after 24 h) and extend (magnitude of the modification in I_D_/I_G_ ratio between the cellular and supernatant compartments). This could be linked to their better iron accessibility. Indeed, TEM observations showed that both S and L-CNT samples presented almost no carbon-based by-products such as amorphous carbon, but did contain Fe-based particles (i.e. catalyst particles) mainly entrapped inside their hollow core. Assuming that the direct consequence of length reduction from L- to S-CNT is a higher amount of CNT extremities for the S-CNT as compared to L-CNT, and even though the total Fe content was similar in S-CNT and L-CNT, the accessibility of Fe nanoparticles encapsulated in S-CNT cores should be higher than in L-CNT. Therefore, during cell exposure, the Fe nanoparticles present in the S-CNT may have been more rapidly, and thus, for a longer time, in contact with the biological medium as compared to those present in the L-CNT. This preferential accountability of iron nanoparticles on the biodegradation of S-CNT is however not visible at a global morphological and structural scale at the time-points studied. Indeed, no noticeable CNT length/diameter reduction or holes occurrence was observed up to 48 h from optical and TEM analyses, as opposed to what Elgrabli et al. recently described [16]. It cannot be excluded, however, the occurrence of such modifications at longer time-points. Our results are difficult to compare with those of the literature, as, as of now, the majority of the studies available in the literature describe modifications occurring in only one batch of CNT [8, 13, 14]. So far, only two studies compared the degradation of different batches of CNT, but none of them utilized pristine CNT, and thus could not be compared to the physico-chemical differences present in the MWCNT of the present study [16, 27].

We are fully aware that our study was performed on a static in vitro system, which represents a far less complex environment than what should happen in vivo in the lung. However, macrophages represent the first line of defense after inhalation of exogenous material, and should, therefore, be the first to recognize and take CNT in charge while inhaled. As such, a large amount of studies combining both in vitro approach using RAW 264.7 macrophages, and in vivo experiments in mice, demonstrates the relevance of the findings obtained in RAW 264.7 cells, and their potential translation to what may occur in vivo [28–31]. We therefore strongly believe that our results are relevant to what occurs in vivo, although this particular issue should be specifically addressed by dedicated studies that were beyond the scope of the present study.

## Conclusion

Our results identify new determinants of CNT biodegradation inside macrophages. This could give new insights for understanding CNT-associated toxicity, and represent important tools to develop safe(r)-by-design nanomaterials.

## Methods

### CNT production

The initial aligned multi-walled CNT powder (at the gram scale) was produced by aerosol-assisted catalytic chemical vapor deposition (CCVD) [32]. This method is based on the catalytic decomposition of liquid hydrocarbons by injecting mixed aerosols containing both the hydrocarbon and the metallic sources which simultaneously and continuously fill the reactor. A solution composed of ferrocene dissolved in toluene (1.25 wt. %) was used to synthesize the nanotube samples at 800 °C under Ar/H_2_ atmosphere (70%/30%). The presence of dihydrogen in the vector of the aerosol allowed to obtain a small external diameter (compared to the 40 nm mean external diameter obtained for a synthesis performed under Ar only), as previously described by Celia Castro et al. [32]. Following this procedure, the sample was formed of aligned CNT carpets covering the reactor walls. The duration of the growth was set at 45 min. Once detached from the reactor walls by scrapping, the precursor sample was treated in de-ionized water (Millipore, 18.2 MΩ.cm), with a dispersing agent (1% biliary salts, composed of 50% sodium deoxycholate (≥98%) and 50% sodium cholate (99%, Acros Organics). An ultrasonic probe Bioblock Vibracell 75043 working at 20 kHz and 375 W in pulse mode (1 s/1 s amplitude, 50% power) was used in order to control the CNT shortening and reach a desired length distribution [33]. Two different durations of ultrasonic treatment (7 h or 5 min) were applied to obtain two distinct groups of CNT: a short and a long group respectively. Both CNT sample powders were then purified at 1000 °C under Ar atmosphere after filtration in order to remove/burn traces of dispersing reagent. Each dry samples was then separated in two sub-groups, treated or not by an acidic solution (75% H_2_SO_4_ and 25% HNO_3_) at 60 °C for 2 h in order to functionalize the nanotubes by grafting oxidized groups on their surface. Finally the different sub-groups were extensively washed with de-ionized water, and final dry samples of CNT were obtained by evaporating water in a fume hood. Ultimately, four distinct groups were obtained (see Additional file 1: Figure S1), with controlled variations in length and surface chemistry: short group (S-CNT), short functionalized group (SF-CNT), long group (L-CNT) and long functionalized group (LF-CNT).

### CNT characterization

#### Optical, scanning electron and transmission electron microscopies

To assess the morphology, structure, and presence of synthesis by-products as well as the diameter and length distributions of the different CNT, samples were observed using optical (Olympus BX60 optical microscope coupled to a color view digital camera, Olympus Corporation, Japan), scanning electron (SEM; Carl Zeiss Ultra 55, field emission gun, Carl Zeiss, Germany) and transmission electron (TEM; Philips CM12 TEM microscope, Philips Research, The Netherlands) microscopes. The morphology and thickness of the CNT precursor carpets were investigated by SEM on sections of aligned CNT carpets fixed on a SEM sample holder with a carbon adhesive tape. To perform TEM analysis, CNT powders were dispersed in ethanol and placed in ultrasonic bath for 1 min (this duration has been chosen to prevent from the introduction of any supplementary morphological/structural modifications). One droplet of this suspension was then deposited on a Cu grid covered with thin carbon film, and grids were observed at 120 kV.

#### Thermogravimetric analysis

To determine the sample initial iron content, the measurement of the remaining iron oxide weight was performed by thermogravimetric analysis (TGA) with a TGA 92–16, 18 Setaram apparatus (Setaram Instrumentation, France) under flowing air at a temperature up to 1000 °C (10 °C/min heating ramp).

#### Raman spectroscopy

Raman spectroscopy was used to evaluate the organization of carbons at the structural (atomic) scale, and especially the degree of order. Carbon organization in CNT samples was analyzed by Raman spectroscopy (Renishaw Invia spectrometer, Renishaw, UK) at an excitation wavelength of 514 nm in the range of 800–3500 cm^−1^. Quantitative Raman parameters were obtained by conventional fitting with a linear baseline and Voigt functions (a combination of Gaussian and Lorentzian functions, the proportion of which is adjusted to each spectrum) using Renishaw Wire 3.2 software. The intensity of the D band and G band were measured from the peak fitting using 1570–1590 cm^−1^ and 1340–1360 cm^−1^ limits for the D and G bands respectively in order to calculate I_D_/I_G_ ratio. Indeed, the D-band is associated with the defect concentration or measure of disorders in the C–C bonds within graphitic materials, while the G-band is associated with in-plane vibrations of C–C bonds and is a measure of graphitization or degree of metallicity of graphitic materials [34]. The characteristic Raman peak intensity ratio ID/IG is a commonly used and useful qualitative and quantitative way of evaluating the structural defects to graphitization or crystallinity ratio in MWCNTs; the degree of order increase when the I_D_/I_G_ ratio decrease (I_D_/I_G_ ratio of graphite = 0) [35].

#### X-ray induced photoelectron spectroscopy

The surface chemical composition of CNT samples was determined by X-ray induced photoelectrons spectroscopy (XPS) using a Kratos Analytical Axis Ultra DLD spectrometer (Kratos Analytical Inc., UK) with monochromatic AlKα X-ray radiation (hν = 1486.6 eV). C1s, O1s and S2p spectra were recorded at a take-off angle of 90° with a 700 μm by 300 μm slot aperture and 20 eV pass energy. The energy scale of the instrument was calibrated by setting Au 4f7/2 = 84.0 eV. Data were acquired with Kratos Analytical Vision 2 software, and peak fitting was performed after Shirley baseline background subtraction using Avantage Thermo Electron software. A Lorentzian/Gaussian ratio of 30% was applied to C1s, O1s and S2p peaks. The atomic sensitivity factors used for semi-quantitative analysis were C1s = 1.0, O1s = 2.93 and S2p = 1.68, relative to C1s = 1.00.

#### Endotoxin contamination of CNT

CNT samples were assessed for endotoxin contamination using the Limulus Amebocyte Lysate assay (Lonza, Switzerland), performed as recommended by the manufacturer.

#### Intrinsic ROS production

The intrinsic production of ROS by CNT was measured in acellular conditions using the properties of φX174 RFI plasmid DNA (Thermo Fisher Scientific, Waltham, MA) [36]. This DNA has the ability to decoil when in presence of ROS. Two hundred and 90 ng of plasmid DNA were incubated with 100 μg/mL CNT for 8 h at 37 °C. The PstI endonuclease (Thermo Fisher Scientific) was used as a positive control. The different forms of DNA in the samples (coiled, decoiled and linearized) were then separated on an agarose gel (0.8%) for 16 h at 30 mV. The intensity of the different DNA bands was quantified, and the ratio of the decoiled and linearized DNA intensity to that of total DNA was calculated.

### Cell culture and exposure to CNT

RAW 264.7 murine macrophages, were purchased from ATCC (Manassas, VA). Cells were cultured in Dulbecco’s modified Eagle medium (DMEM) supplemented with 10% heat-inactivated fetal calf serum and antibiotics (streptomycin, 10 mg/mL; penicillin G, 10,000 IU/mL) at 37 °C in a humidified atmosphere of 5% CO_2_/95% air. Cells were exposed for 6, 24 or 48 h to 50 μg/mL CNT prepared by dispersion of the dry material sample in serum-free cell culture medium. For homogenization purpose, the CNT suspensions were sonicated and vortexed just before cell exposure. Culture medium alone was used as a control. In a subset of experiments, cells were treated with Concanamycin (Conca, 10 nM; Sigma-Aldrich) or Lysosensor DND-189 (10 μM; Thermo Fisher Scientific) 2 h prior to the end of CNT exposure.

For Raman spectroscopy analysis, at the end of cell exposure, the cell supernatant was recovered, and cells were washed three times in PBS. This washing solution was added to the supernatant, centrifuged at 6000 rpm for 15 min and washed 3 times with 10 mL of ultrapure water to obtain a CNT pellet (SN fraction). To obtain the CNT pellet from the Cell fraction, cells attached to the cell culture dish were also washed 3 times with 10 mL of ultrapure water and centrifuged at 6000 rpm for 15 min (Cell fraction). A few drops of each CNT pellet (obtained from Cell and SN fractions) were deposited on a glass slide and dried in an oven at 100 °C for further analysis by Raman spectroscopy.

### Transmission electron microscopy

Samples were analyzed using TEM (JEOL microscope, Japan) to observe cells and cell components exposed to 50 μg/mL CNT for 6 h. Ultra-thin sections (90 nm) of the cell samples were prepared as previously described [37] and deposited on a Cu grid covered with thin carbon film. Grids were observed at 80 kV.

### Lysosensor assay

RAW 264.7 cells were exposed to 10 μg/mL CNT for 6, 24 or 48 h, and Lysosensr DND-189 (10 μM, Lifetechnologies) was added for the last 2 h. After exposure, the cells were fixed and the fluorescence images were digitally acquired on a Zeiss Axio Imager M2 (Carl Zeiss). Fluorescence intensity was quantified (arbitrary units) in at least 20 different cells per condition.

### Statistical analysis

Otherwise mentioned, each value is given as the mean ± standard error of the mean (SEM) of at least 3 experiments performed in triplicate. Data were analyzed with the GraphPad Prism 5.01 software (La Jolla, CA). Comparisons between multiple groups were performed using Kruskall–Wallis’ non-parametric analysis of variance test followed, when a difference was detected, by two-by-two comparisons with Dunn’s multiple comparisons test. *P*-values <0.05 were considered significant.

## Additional files

Additional file 1: Figure S1. Experimental set-up for CNT synthesis. Experimental set-up for CNT synthesis leading to the obtainment of 4 final batches of CNT: S-, SF-, L-, and LF-CNT. Abbreviations as in Fig. 1. (PDF 49 kb)
Additional file 2: Figure S2. Raman spectra. Raw Raman spectra obtained from CNT recovered in macrophages exposed to the different CNT for 48 h (Cell fraction). Abbreviations as in Fig. 1. (PDF 32 kb)
Additional file 3: Figure S3. Lysosomal activity assessment. Lysosensor assay in RAW 264.7 macrophages exposed to CNT for 24 h (Panel **a**) or 48 h (Panel **b**). Scale bar: 10 μm. Abbreviations as in Fig. 1. Panel **c**: quantification (arbitrary units) of fluorescence intensity at 6 h time point. Panel **d**: quantification (arbitrary units) of fluorescence intensity at 24 h time point. b quantification (arbitrary units) of fluorescence intensity at 48 h time point. *: *p* < 0.05 vs Control condition. (PDF 587 kb)

## Abbreviations

CCVD: Catalytic chemical vapor deposition
CNT: Carbon nanotubes
EPO: Eosinophil peroxidase
HRP: Horseradish peroxidase
MPO: Myelo-peroxidase
MWCNT: Multiwalled carbon nanotubes
NOX: NAD(P)H oxidase
OM: Optical microscopy
SEM: Scanning electron microscopy
SWCNT: Singlewalled carbon nanotubes
TEM: Transmission electron microscopy
TGA: Thermogravimetric analysis
XPS: X-ray-induced photoelectron spectroscopy
