# Supplementary figures and images for: Early signs of multi-walled carbon nanotbues degradation in macrophages, via an intracellular pH-dependent biological mechanism; importance of length and functionalization
Source: Part Fibre Toxicol. 2016 Nov 24;13:61. doi: 10.1186/s12989-016-0175-z (PMC5122009; doi:10.1186/s12989-016-0175-z)

Figure S1

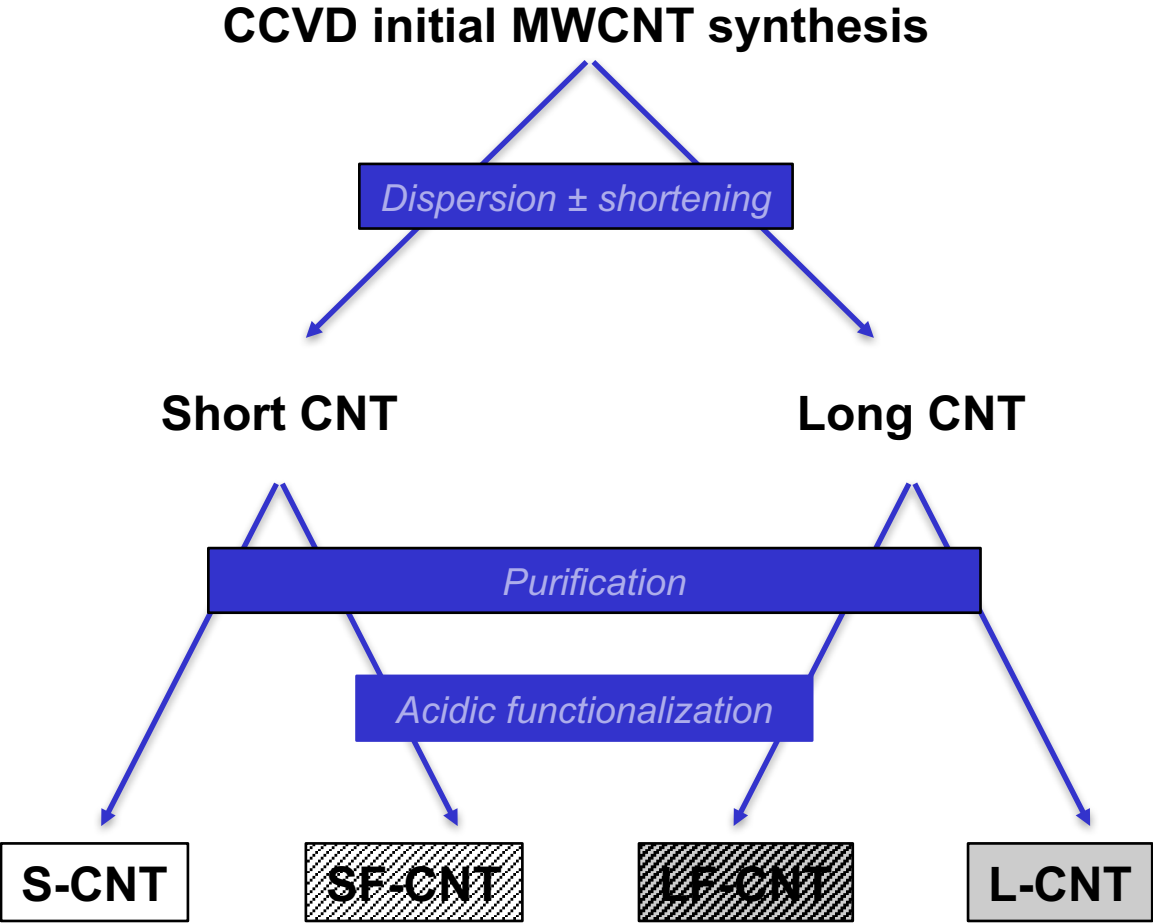

Supplement: Additional file 1: Figure S1. — Experimental set-up for CNT synthesis. Experimental set-up for CNT synthesis leading to the obtainment of 4 final batches of CNT: S-, SF-, L-, and LF-CNT. Abbreviations as in Fig. 1. (PDF 49 kb) [file 12989_2016_175_MOESM1_ESM.pdf]

Figure S2

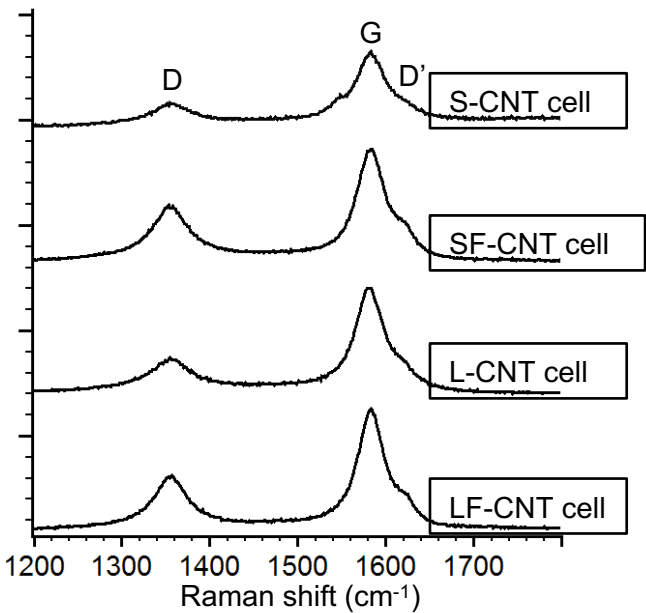

Supplement: Additional file 2: Figure S2. — Raman spectra. Raw Raman spectra obtained from CNT recovered in macrophages exposed to the different CNT for 48 h (Cell fraction). Abbreviations as in Fig. 1. (PDF 32 kb) [file 12989_2016_175_MOESM2_ESM.pdf]

Figure S3

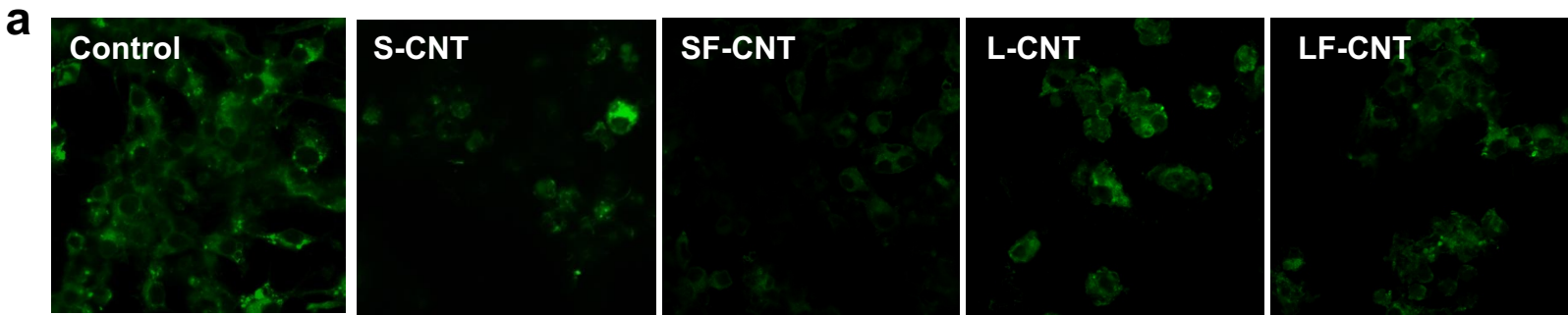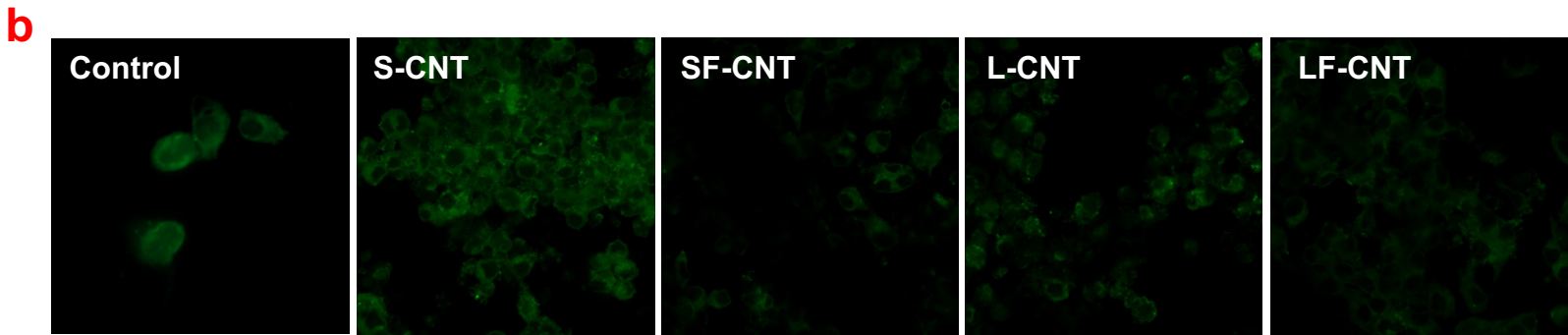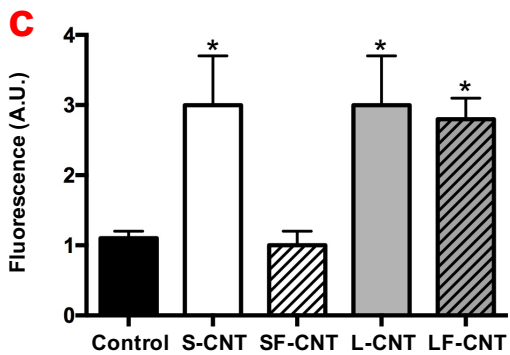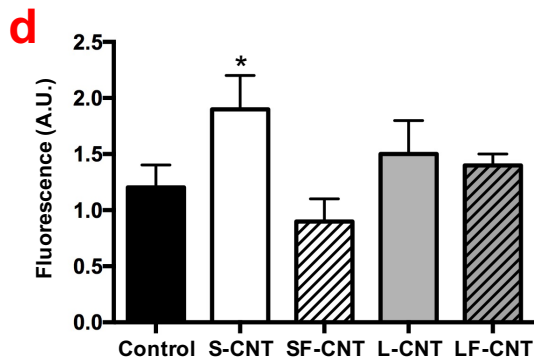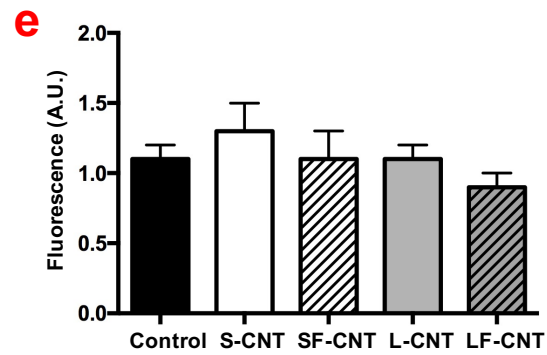

Supplement: Additional file 3: Figure S3. — Lysosomal activity assessment. Lysosensor assay in RAW 264.7 macrophages exposed to CNT for 24 h (Panel a) or 48 h (Panel b). Scale bar: 10 μm. Abbreviations as in Fig. 1. Panel c: quantification (arbitrary units) of fluorescence intensity at 6 h time point. Panel d: quantification (arbitrary units) of fluorescence intensity at 24 h time point. b quantification (arbitrary units) of fluorescence intensity at 48 h time point. *: p < 0.05 vs Control condition. (PDF 587 kb) [file 12989_2016_175_MOESM3_ESM.pdf]
